# Impact of Interleukin 28B Genotype on the Virological Responses in Chronic Hepatitis C Treatment

**DOI:** 10.14740/gr629e

**Published:** 2014-12-27

**Authors:** Bilgehan Aygen, Orhan Yildiz, Sila Akhan, Ozgur Gunal, Serpil Taheri, Gokmen Zararsiz, Murat Sayan, Aydin Rustemoglu, Elif Sargin Altinok

**Affiliations:** a Department of Infectious Diseases and Clinical Microbiology, Medical School of Erciyes University, Kayseri, Turkey; b Kocaeli University Medical Faculty Infectious Diseases and Clinical Microbiology, Kocaeli, Turkey; c Department of Infectious Diseases and Clinical Microbiology, Medical School of Gaziosmanpasa University, Tokat, Turkey; d Erciyes University Betul Ziya Eren Genome and Stem Cell Center, Kayseri, Turkey; e Department of Biostatistics, Medical School of Erciyes University, Kayseri, Turkey; f Department of Infectious Diseases and Clinical Microbiology, Medical School of Kocaeli University, Kocaeli, Turkey; g Gaziosmanpasa University Medical Faculty, Department of Medical Biology, Tokat, Turkey

**Keywords:** Chronic hepatitis C, Interleukin 28B, Polymorphism, Genetic, Pegylated interferon

## Abstract

**Background:**

Interleukin (IL) 28B single nucleotide polymorphisms may play a role in the clearance of hepatitis C virus (HCV). We aimed to evaluate the treatment response of chronic HCV infection patients to pegile interferon (pegIFN) and ribavirin treatment with regard to IL28B rs12979860 C/T polymorphism.

**Methods:**

A total of 186 patients (mean age, 55.6 ± 10 years; 65.1% female) who underwent pegIFN and ribavirin treatment for chronic HCV infection were studied. We analyzed demographics, HCV genotype, baseline alanine aminotransferase (ALT) levels, histopathological data, viral load before treatment and at 4, 12, 24, 48, and 72 weeks from the treatment start, and IL28B genotype. IL28B polymorphism was genotyped using polymerase chain reaction based restriction fragment length polymorphism (PCR-RFLP) in all the subjects.

**Results:**

One hundred forty-five (86.8%) patients were infected with viral genotype 1b, and 13.2% were infected with viral genotype 4. The rates of C/C, C/T, and T/T genotypes were 22.6%, 52.7%, and 24.7% respectively. The percentage of patients with a viral load over 400,000 IU/mL was higher in the C/T group (P = 0.020). Of the patients, 44.6% provided sustained virological response (SVR) to pegIFN and ribavirin combination treatment. The frequency of T allele was 41% in patients with SVR, whereas 59% patients provided no response (P < 0.001). SVR was obtained in 66.7%, 42.9%, and 28.3% of CC, CT, and TT groups (P = 0.001). The rates of rapid virological response (RVR), early virological response (EVR), end-of-treatment response (ETR), and SVR were higher in the CC group than other groups (P = 0.216, P < 0.001, P = 0.001, P = 0.001, respectively). The relapse and null response (NR) rates were higher in TT group and partial response rate (PR) was higher in CT group.

**Conclusions:**

IL28B rs12979860 C/T gene polymorphism affects the response to antiviral treatment in the patients with chronic HCV genotypes 1b and 4 infections.

## Introduction

There are several factors that affect the progression of hepatitis C virus (HCV) infection and its response to treatment in chronic cases [1-4]. In multiple genome-wide association studies, a single nucleotide polymorphism of the interleukin (IL) 28B gene, 3 kb upstream (rs12979860 C/T), encoded by interferon (IFN) lambda-3, had a substantial role in the disappearance of HCV infection spontaneously or with treatment [5]. IL28B rs12979860 is the primary factor contributing to recovery from infection [6-8]. In some recent studies, it has been observed that IL28B gene polymorphism increases almost two-fold response to treatment [9, 10]. The IL28B genotype is a strong predictor of SVR in treatment protocols that include a protease inhibitor or in IFN/ribavirin combination treatments [11-13].

The present study aimed to evaluate the characteristics and response to treatment of Turkish patients who underwent pegile (peg) IFN and ribavirin for chronic hepatitis C (CHC) with respect to IL28B rs12979860 C/T polymorphism.

## Methods

### Patients

One hundred eighty-six patients with CHC (121 female and 65 male) from three university hospitals located in two different geographical regions who underwent pegIFN and ribavirin treatment and were followed up regularly between April 2011 and April 2013 were involved in this retrospective study. The mean age was 55.6 ± 10 years (age range, 24 - 77 years). CHC was diagnosed in cases with compensated disease through liver biopsy, normal or high liver enzymes, positive anti-HCV, and positive HCV-RNA. Patients with other chronic liver diseases, positive hepatitis B surface antigen (HBsAg), and human immunodeficiency virus (HIV) infection were excluded from the study. The liver biopsies percutaneously obtained were evaluated using the Ishak scoring system [14], which categorizes fibrosis with a score of 0 - 2 as mild, 3 - 4 as moderate, and 5 - 6 as severe [15].

### Evaluation of treatment protocol, follow-up, and treatment responses

Eighty-seven patients were administered pegIFN alpha-2a plus ribavirin, and 99 were administered pegIFN alpha-2b plus ribavirin at standard doses, as follows: pegIFN alpha-2a 180 µg/week + ribavirin (1,000 mg/day for patients with body weight ≤ 75 kg; 1,200 mg/day for patients with body weight > 75 kg); or pegIFN alpha-2b (1.5 µg/kg/week) + ribavirin (800 mg/day for patients with body weight < 65 kg; 1,000 mg/day for patients with body weight 65 - 85 kg; 1,200 mg/day for patients with body weight 85 - 105 kg; 1,400 mg/day for patients with body weight > 105 kg) [16]. Length of treatment period was determined in accordance with the regulations of National Health Practices Statement of the Health Ministry. According to this official statement, the duration of treatment for genotypes 1 and 4-infected patients is 48 week, and treatment cannot be continued for more than 16 weeks for the patients not displaying a decrease of 2 log_10_ in HCV-RNA level in the 12th week of treatment. For patients with a 2 log_10_ decrease in HCV-RNA level after 12 weeks, if HCV RNA positivity still goes on in the 24th week, the length of treatment can be up to 28 weeks.

Clinical evaluation and laboratory tests of the patients undergoing treatment were performed in the first, second, and fourth week of treatment, then monthly until the end of the treatment, and 3 and 6 months after the end of the treatment. PegIFN and ribavirin doses were modified on the basis of weights, symptoms, and laboratory findings of the patients. Quantitative HCV-RNA measurement was studied before the treatment in the fourth, 12th, 24th, and 48th week of treatment, and 24 weeks after the treatment. The responses to the treatment were described according to guidelines [13, 16].

For all patients, demographics, body mass indices (BMI), alanine aminotransferase (ALT) and viral genotypes, liver biopsy necroinflammation, and fibrosis results, which were evaluated on the basis of the Ishak scoring system, and HCV-RNA results (before treatment and 4, 12, 24, 48, and 72 weeks after treatment) were recorded on private forms designed for the study. Liver biopsy was performed in 152 of 186 patients. All subjects provided written informed consent for both treatment and genetic analysis. The study was approved by the Ethics Committee for Clinical Research at Kocaeli University, which conforms to protocols in accordance with the Declaration of Helsinki (decision number: 2013-39).

### Blood samples and laboratory tests

Routine biochemical tests were performed from venous blood samples with an automated device and HBsAg, anti-HCV, and anti-HIV antibodies examined using an enzyme immunoassay method (anti-HCV and anti-HIV: Architect System, Abbott Laboratories, IL, USA, HBsAg: Elecsys Systems, Roche Diagnostic, Mannheim, Germany). Quantitative HCV-RNA measurement was performed using real-time polymerase chain reaction (PCR; COBAS Ampliprep/COBAS TaqMan 48, Roche Molecular Systems, Mannheim, Germany and Bosphore HCV Quantification v2, Anatolia Geneworks, Istanbul, Turkey), and the HCV genotype was determined using the pyrosequencing method, according to the manufacturer’s instructions. After DNA isolation from blood samples, they were stored at -80 °C.

### Detection of the IL28B rs12979860 C/T polymorphism

Genotyping for the IL28B rs12979860 C/T polymorphism was performed by a PCR-based restriction fragment length polymorphism assay. Genomic DNA was extracted from whole blood samples using the QIAamp DNA blood mini kit (Qiagen, Hilden, Germany), according to manufacturer’s instruction. Using purified genomic DNA, a 139-bp product was amplified with the following primers: forward primer 5’-CCAGGGCCCCTAACCTCTGCA-3’ and the reverse primer 5’-GGGAGCGCGGAGTGCAATTCA-3’. Amplification was performed in a total volume of 50 μL containing 10 mmol/L Tris-HCl (pH 8.3), 50 mmol/L KCl, 0.01% Tween-20, 0.2 mmol/L deoxyribonucleotides, 2 - 4 pmol of each primer, 2.0 mmol/L MgCl_2_, 0.5 units hot-start Taq DNA polymerase (Thermo Taq, Thermo Scientific, Pittsburgh, PA, USA), and approximately 10 ng of genomic DNA. The thermal protocol for amplification included 35 cycles of denaturation at 95 °C for 60 s, annealing at 62 °C for 60 s, and elongation at 72 °C for 60 s. Ten microliters of the amplicons were digested with 1 unit *Bst*UI (New England Biolabs, Hitchin, UK) in a total volume of 20 μL at 37 °C overnight. The fragments were resolved by 4% agarose electrophoresis after staining with ethidium bromide. A band of 139 bp indicated the TT genotype, 109 bp indicated the CC genotype, and 139 + 109 bp indicated the CT genotype [17].

### Statistical analysis

Histogram, q-q plots, and Shapiro-Wilk’s test were used to assess data normality. A logarithmic transformation was applied (base 10) to baseline viral load variables because of its highly skewed distribution and discrete count data type. To compare the differences between groups, one-way analysis of variance (ANOVA) and Kruskal-Wallis test were used for continuous variables and Pearson’s Chi-square analysis was used for categorical variables. In addition, Siegel-Castellan test was performed for multiple comparisons. Odds ratios (ORs) were calculated with 95% confidence intervals (CIs), and values were expressed as frequencies and percentages, means and standard deviations (SDs), medians and interquartile ranges, or geometric means and 95% CIs. Analysis was conducted using R 3.0.2 software (www.r-project.org), considering a P value of < 0.05 as statistically significant.

## Results

The demographic characteristics of 186 CHC patients are shown in Table 1. When the IL28B genotype was assessed in patients with CHC, the C/C genotype was identified in 22.6%, the C/T genotype in 52.7%, and the T/T genotype in 24.7% patients. The characteristics of patients are summarized in Table 2 on the basis of the IL28B genotype subgroups. The frequency of patients with a viral load of ≥ 400,000 IU/mL was higher in the C/T group (P = 0.020), and baseline ALT was higher in the TT group (P = 0.006). The median liver fibrosis score was 1 for patients with C/C and T/T genotypes and 2 for those with the C/T genotype (P = 0.023). There was no statistically significant difference between IL28B subgroups in terms of mean age (P = 0.147), gender distribution (P = 0.571), mean BMI (P = 0.446), genotype 1b or genotype 4 distributions (P = 0.091), the mean baseline HCV-RNA level (P = 0.503), and median liver necroinflammation (P = 0.669).

**Table 1:** Characteristics of Patients With Chronic Hepatitis C

Gender, n (%)	
Female	121 (65.1)
Male	65 (34.9)
Age (years), mean ± SD	55.6 ± 10
BMI (kg/m^2^), mean ± SD	27.3 ± 4.6
Normal weight (< 25 kg/m^2^), n (%)	55 (29.6)
Overweight (25-29.9 kg/m^2^), n (%)	95 (51.1)
Obese (≥ 30 kg/m^2^), n (%)	36 (19.4)
Viral load at baseline (IU/mL), geometric mean (95% CI)^a^	854,053 (663,578 - 1,087,754)
< 400,000 (IU/mL), n (%)	56 (30.1)
≥ 400,000 (IU/mL), n (%)	130 (69.9)
Genotype, n (%)^b^	
Genotype 1b	145 (86.8)
Genotype 4	22 (13.2)
Baseline ALT levels (IU/L), median (first to third quartiles)	49.0 (34.0 - 69.0)
Liver necroinflammation^c^, median (first to third quartiles)	5.5 (4.0 - 8.0)
Liver fibrosis^c^, median (first to third quartiles)	2.0 (1.0 - 3.0)
Distribution of liver fibrosis, n (%)	
Mild (Ishak staging score 0 - 2)	107 (70.5)
Moderate (Ishak staging score 3 - 4)	35 (23.0)
Severe (Ishak staging score 5 - 6)	10 (6.5)

BMI: body mass index; ALT: alanin aminotransferase; SD: standart deviation; CI: confidence interval. ^a^Logarithmic transformation (base 10) was applied. Confidence intervals of geometric mean are computed using 1,000 bootstrap samples. ^b^Genotypes were determined in 167 patients. ^c^152 patients with liver biopsy, evaluated based on the Ishak score.

**Table 2:** Patients Characteristics in Each IL28B Genotype Subgroup

Variables	C/C (n = 42)	C/T (n = 98)	T/T (n = 46)	P
Age (years), mean ± SD	53.3 ± 11.2	55.7 ± 9.2	57.5 ± 10.2	0.147
Male, n (%)	17 (40.5)	31 (31.6)	17 (37)	0.571
BMI (kg/m^2^), mean ± SD	26.9 ± 4.3	27.7 ± 4.7	26.9 ± 4.6	0.446
Viral load at baseline (IU/mL), geometric mean (95% CI)^a^	987,812 (571,056 - 1,811,751)	905,147 (648,435 - 1,296,846)	660,740 (406,456 - 1,055,059)	0.503
Patients with baseline viral load < 400,000 IU/mL, n (%)	18 (42.9)	21 (21.4)	16 (34.8)	0.020
Genotype 1b^b^, n (%)	37 (97.4)	73 (83.9)	35 (83.3)	0.091
Genotype 4^b^, n (%)	1 (2.6)	14 (16.1)	7 (16.7)	
Baseline ALT levels (IU/L), median (first to third quartiles)	50.0 (34.0 - 83.0)^xy^	44.0 (29.0 - 59.0)^x^	57.5 (37.0-87.0)^y^	0.006
Liver necroinflammation^c^, median (first to third quartiles)	6.0 (4.0 - 6.0)	5.0 (4.0 - 8.0)	6.0 (4.0 - 8.0)	0.669
Liver fibrosis^c^, median (first to third quartiles)	1.0 (1.0 - 2.0)^x^	2.0 (1.0 - 3.0)^y^	1.0 (1.0 - 3.0)^xy^	0.023
Distribution of liver fibrosis, n (%)				
Mild (Ishak staging score 0 - 2)	25 (83.3)^d^	53 (63.9)^e^	29 (74.4)^f^	0.236
Moderate (Ishak staging score 3 - 4)	5 (16.7)^d^	23 (27.7)^e^	7 (17.9)^f^	
Severe (Ishak staging score 5 - 6)	0 (0.0)^d^	7 (8.4)^e^	3 (7.7)^f^	

BMI: body mass index; ALT: alanin aminotransferase; SD: standart deviation; CI: confidence interval. ^a^Logarithmic transformation (base 10) was applied. Confidence intervals of geometric mean are computed using 1,000 bootstrap samples. ^b^Genotypes were determined in 167 patients. ^c^Cases with liver biopsy were evaluated based on Ishak score. ^d^30 patients. ^e^83 patients. ^f^39 patients. Different superscripts in a row (x, y) indicate statistically significant difference between groups.

A total of 44.6% patients provided SVR to pegIFN and ribavirin combination treatment, while 55.4% (103 patients) did not. The frequency of T allele for the patients providing SVR was 41%, but this rate was 59% for the patients with no response (P < 0.001). SVR was obtained from 66.7% of the CC genotype, 42.9% of the CT genotype, and 28.3% of the TT genotype (P = 0.001) (Table 3). The responses to the treatment are shown in Table 4. The values of RVR (P = 0.216), cEVR (P < 0.001), EVR (P < 0.001), ETR (P = 0.001), and SVR (P = 0.001) were higher in the CC genotype than those in other genotype groups. However, there was no statistically significant difference in terms of RVR. Partial response (PR) was observed to be higher in the CT genotype (P = 0.443), whereas the rates of relapse and null response (NR) were higher in the TT genotype group (P = 0.400, P = 0.001).

**Table 3:** Distribution of IL28B rs12979860 C/T Genotypes and Alleles in Patients With Sustained Virologic Response and Non-Responder

	IL28B	SVR (n = 83), n (%)	Non-response (n = 103), n (%)	P	OR (95% CI)
rs12979860 alleles	C	98 (59)	84 (41)	< 0.001	-
	T	68 (41)	122 (59)	< 0.001	
rs12979860 genotypes	CC	28 (66.7)	14 (33.3)	0.001	1
	CT	42 (42.9)	56 (57.1)		0.38 (0.18 - 0.80)
	TT	13 (28.3)	33 (71.7)		0.20 (0.08 - 0.49)

SVR: sustained virologic response; OR: odds ratio; CI: confidence interval.

**Table 4:** Relationship Between IL28B Genotype and Response to Treatment Rates

Variables, n (%)	C/C (n = 42)	C/T (n = 98)	T/T (n = 46)	P
RVR	23 (54.8)	39 (41.9)^a^	18 (39.1)	0.216
cEVR	37 (88.1)	57 (58.2)	22 (47.8)	< 0.001
EVR (cEVR + pEVR)	41 (97.6)	71 (72.4)	29 (63.0)	< 0.001
ETR	38 (90.5)	61 (62.2)	26 (56.5)	0.001
SVR	28 (66.7)	42 (42.9)	13 (28.3)	0.001
Relapse	11 (26.2)	20 (20.4)	14 (30.4)	0.400
PR	1 (2.4)	8 (8.2)	3 (6.5)	0.443
NR	1 (2.4)	25 (25.5)	16 (34.8)	0.001
Breakthrough	1 (2.4)	3 (3.1)	0 (0.0)	0.495

RVR: rapid virological response; cEVR: complete early virological response; EVR: early virological response; pEVR: partial early virological response; ETR: end-of-treatment response; SVR: sustained virological response; PR: partial response; NR: null response. ^a^Five patients were not evaluated for RVR.

## Discussion

Chronic HCV infection is a serious problem in Turkey. Although the TURKHEP study reported a nationwide seroprevalence of 1%, there are some regions where this rate is as high as 3.1% [18, 19], and prevalence increases after age 50 years [20]. In Turkey, the endemic HCV genotype is genotype 1b, and recently, genotype 4 infection has been more frequently observed, possibly because of immigration [21]. Both genotypes belong to the group, in which SVR to the drugs used for CHC is low. Therefore, before initiating treatment, it is important to predict the response of the patient.

Genome-wide association has mapped different phenotypes or characteristics for several single nucleotide polymorphisms on all chromosomes [22]. It has been indicated that the IL28B rs12979860 C/T polymorphism plays an important role in the response of CHC to the treatment and the spontaneous clearance of the virus [5, 6, 9, 10, 23]. It was reported that, in African or European populations, HCV spontaneous clearance was three-fold higher in patients with the IL28B rs12979860 C/C genotype compared with those with the C/T and T/T genotypes [6]. El-Awady et al [24] reported that IL28B C/C genotype protects against chronic infection progression by spontaneous clearance during the acute phase. In other studies, the C/C genotype played a major role in viral clearance caused by treatment [25-27].

In the present study, 22.6% CHC patients were of the C/C, 52.7% cases were of the C/T, and 24.7% were of the T/T genotype. In a study, the distribution of the IL28B rs12979860 genotypes was examined in HCV-genotype-4-infected patients. The prevalence of the C/C genotype was 30.7%, that of the C/T genotype was 49.5%, and that of the T/T genotype was 19.8% [28]. In a study performed with 871 Caucasian patients, 39% patients with HCV infection were of the C/C genotype, 49% were of the C/T genotype, and 12% were of the T/T genotype [29]. In another study performed in Romania, these rates were 24.3%, 62.6%, and 13.1%, respectively [11]. C/T was the most prevalent genotype in our study, which is similar to the results of these studies. In our CHC patients, the frequency of the C/C genotype was lower than those previously reported in American and European HCV-infected patients [6, 9, 30, 31]. The frequency of the C/C genotype for HCV-infected patients in Europe was reported as 38% [9] and 45% [31].

When characteristics of the patients with CHC were evaluated on the basis of IL28B genotype subgroups, the frequency of patients with a viral load of ≥ 400,000 IU/mL was highest in the C/T group (P = 0.021). The median ALT level was higher in the TT genotype (P = 0.006). Montes-Cano et al [31] reported that in 284 CHC patients, there was no significant difference between the patients in the C/C and C/T + T/T groups with regard to viral load or in gender distribution and infection age; however, the ALT level was higher in the C/C group (P = 0.006). Sporea et al [11] determined that the baseline viral load was significantly lower in the T/T group than other genotypes, and the frequency of patients with a viral load of > 600,000 IU/mL was highest for the C/C genotype (80.8%).

In our study, 86.8% patients had genotype 1b infection and 13.2% had genotype 4 infection. The liver fibrosis score was significantly higher in subjects with the C/T genotype compared with other genotypes (P = 0.023), although there were no differences in necroinflammation among the groups. In the study by Falleti et al [15], the frequency of genotype 1 infection was 28.1%, that of genotype 2 was 38.2%, that of genotype 3 was 42.3%, and that of genotype 4 - 5 was 36.6% for the C/C group. In the same study, the necroinflammation score in the C/C group was significantly higher than that in the C/T + T/T group. The number of patients with severe hepatitis was higher in the T/T group, while the number of patients with mild hepatitis was higher in the C/C group. In the study by Montes-Cano et al [31], for the group with the IL28B C/C genotype, the rate of HCV infection with genotype 1 was 39.1% and the frequency of the other genotypes was 66.7%. Based on liver biopsy results, a fibrosis score of 0 - 2 was observed in 46.5% and a score of 3 - 4 was observed in 43.2% patients with the C/C genotypes. In the C/C + C/T group, a fibrosis score of 0 - 2 was observed in 53.5% and a score of 3 - 4 in 56.8% patients. The differing results in our study may be because the number of patients was low, the number of patients in the C/T group was higher than those in the other groups, and 70.5% had mild liver disease.

In CHC patients, there is a relationship between IL28B genotype and SVR to pegIFN alpha and ribavirin treatment. In the studies conducted in Asia, high rate of SVR in CHC patients can be explained with the fact that IL28B genotype of these patients is generally C/C [32, 33]. Moreover, Ge et al [9] indicated that the rate of SVR was two-fold higher in the C/C genotype than in the T/T genotype (95% CI: 1.8 - 2.3).

In our study, general rate of SVR given to the pegIFN alpha and ribavirin combination treatment was 44.6%, but in the C/C, C/T, and T/T genotypes, this rate was observed to be 66.7%, 42.9%, and 28.3%, respectively (P = 0.001). RVR rates were 54.8% in the C/C genotype, 41.9% in the C/T genotype, and 39.1% in the T/T genotype. The rates of ETR were 90.5% in the C/C genotype, 62.2% in the C/T genotype, and 56.5% in the T/T genotype. With regard to the rates of PR, there was no significant difference among the groups. However, the rate of the patients with NR was significantly higher in the T/T genotype than the C/C and C/T genotypes (P = 0.001). In a previous study, genotype 1-infected 1,171 Caucasian, 300 African-American, and 116 Hispanic CHC patients were evaluated, and the percentages of SVR were observed to be 69% for the C/C genotype, 33% for the C/T genotype, and 27% for the T/T genotype groups [34]. In this study, the rates of RVR were determined as 28%, 5% and 5%, and the rates of ETR were 92%, 56%, and 51% respectively. Our ETR and SVR rates were similar to the rates of this study, but our RVR ratios were higher for all of the genotype groups. In a multicenter study conducted in Germany, the SVR rates of patients with C/C, C/T, and T/T genotypes were observed to be 85%, 58%, and 46%, respectively [35]. In another study in Romania, the SVR rates were 73.1%, 40.9%, and 57.1% according to the genotypes [11]. The rates of SVR obtained from the C/C and T/T genotypes in this study were similar to those reported by Thompson et al [34]. Stattermayer et al [36] observed that the rate of RVR was 38.3% and the rate of SVR was 79.1% for the genotype 1-infected patients with CC allele. In addition, these rates were respectively 76.5% and 85.3% for the genotype 4-infected patients with CC allele. Therefore, an EVR to pegIFN and ribavirin is more likely to be observed among carriers of the C/C IL28B polymorphism, which may underlie their high rate of SVR, and that IL28B genotype determination and the presence of an RVR may be used in future studies of patients with HCV genotypes 1 or 4.

In our study, it was observed that the ratio of cEVR + pEVR was 97.6% for the C/C genotype group, 72.4% for the C/T genotype, and 63% for the C/C genotype. Sporea et al [11] indicated that the rates of EVR in genotype 1-infected 107 patients were 100%, 89.5%, and 85.7%, respectively. The same rates in the study of Thompson et al [34] were 97%, 72%, and 68%, respectively. The rates of relapse in our study were 30.4% for the T/T genotype, but 26.2% and 20.4% for the C/C and C/T genotypes. In another study, the rates of relapse were close to each other, i.e., as 13%, 19%, and 21% for the C/C, C/T, and T/T genotype groups, respectively [35].

In our study, the frequency of T allele was lower in the patients with SVR than in those with no response (41% vs. 59%, P < 0.001). In the study of Pasha et al [28], the SVR ratios of genotype 4-infected Egyptian patients were observed to be 38.2% for those having C/C genotype, 50% for those having C/T genotype, and 11.8% for those having T/T genotype. The percentage of T allele was 36.8% in the group with SVR. In another study conducted in Egypt, based on the C/C, C/T, and T/T genotypes, the rates of SVR were observed to be 71.4%, 20.6%, and 7.9%, respectively [37]. In this study, the rate of T allele was 18.2% for the ones with SVR and 47.2% for the ones with no response.

In conclusion, our findings suggest that the antiviral treatment response in CHC is connected with the IL28B rs12979860 C/T gene polymorphism. Among the HCV genotype 1b- and 4-infected patients, the rates of RVR, EVR, ETR and SVR obtained from pegIFN and ribavirin treatment were higher in the patients having IL28B C/C genotype than those with C/T and T/T genotypes. These findings may help clinicians decide on beginning both pegIFN alpha and ribavirin treatment and triple antiviral treatment, including protease inhibitor, the cost of which is higher than dual treatment. Further comprehensive studies should examine the effects of the IL28B genotype subgroups on the prognosis of the disease and the response to treatment for CHC patients.

## References

[1] Aygen B, Yildiz O, Caylan R, Koksal I, Saltoglu N, Tasova Y (2004). Combination of interferon induction therapy and ribavirin in chronic hepatitis C patients: results of a multicenter clinical trial. Viral Hepatitis Journal.

[2] Yildiz O, Aygen B, Alp E, Gokahmetoglu S, Soyuer I (2008). Retrospective evaluation of different antiviral treatments for chronic hepatitis C infection. Flora the Journal Infectious Diseases and Clinical Microbiology.

[3] Aygen B, Yildiz O, Bostanci F, Ozbakir O, Gokahmetoglu S (2004). A comparison of interferon-alpha-2b induction therapy with interferon-alpha-2b plus ribavirin combination therapy for the treatment of chronic hepatitis C. Flora the Journal Infectious Diseases and Clinical Microbiology.

[4] Demiraslan H, Aygen B, Yildiz O, Soyuer I, Gokahmetoglu S (2008). A comparison of interferon α-2a plus ribavirin combination therapy with peginterferon α-2a plus ribavirin combination therapy for treatment of chronic hepatitis C. Viral Hepatitis Journal.

[5] Rauch A, Kutalik Z, Descombes P, Cai T, Di Iulio J, Mueller T, Bochud M (2010). Genetic variation in IL28B is associated with chronic hepatitis C and treatment failure: a genome-wide association study. Gastroenterology.

[6] Thomas DL, Thio CL, Martin MP, Qi Y, Ge D, O'Huigin C, Kidd J (2009). Genetic variation in IL28B and spontaneous clearance of hepatitis C virus. Nature.

[7] Fonseca-Coronado S, Vaughan G, Cruz-Rivera MY, Carpio-Pedroza JC, Ruiz-Tovar K, Ruiz-Pacheco JA, Escobar-Gutierrez A (2011). Interleukin-28B genotyping by melt-mismatch amplification mutation assay PCR analysis using single nucleotide polymorphisms rs12979860 and rs8099917, a useful tool for prediction of therapy response in hepatitis C patients. J Clin Microbiol.

[8] Lagging M, Askarieh G, Negro F, Bibert S, Soderholm J, Westin J, Lindh M (2011). Response prediction in chronic hepatitis C by assessment of IP-10 and IL28B-related single nucleotide polymorphisms. PLoS One.

[9] Ge D, Fellay J, Thompson AJ, Simon JS, Shianna KV, Urban TJ, Heinzen EL (2009). Genetic variation in IL28B predicts hepatitis C treatment-induced viral clearance. Nature.

[10] Grebely J, Petoumenos K, Hellard M, Matthews GV, Suppiah V, Applegate T, Yeung B (2010). Potential role for interleukin-28B genotype in treatment decision-making in recent hepatitis C virus infection. Hepatology.

[11] Sporea I, Popescu A, Curescu M, Sirli R, Dan I, Goldis A, Gradinaru O (2011). The Correlation of Il28B Genotype With Sustained Virologic Response In Romanian patients With Chronic Hepatitis C. Hepat Mon.

[12] Ghany MG, Nelson DR, Strader DB, Thomas DL, Seeff LB (2011). An update on treatment of genotype 1 chronic hepatitis C virus infection: 2011 practice guideline by the American Association for the Study of Liver Diseases. Hepatology.

[13] Omata M, Kanda T, Yu ML, Yokosuka O, Lim SG, Jafri W (2012). APASL consensus statements and management algorithms for hepatitis C virus infection. Hepatol Int.

[14] Ishak K, Baptista A, Bianchi L, Callea F, De Groote J, Gudat F, Denk H (1995). Histological grading and staging of chronic hepatitis. J Hepatol.

[15] Falleti E, Bitetto D, Fabris C, Cussigh A, Fornasiere E, Cmet S, Fumolo E (2011). Role of interleukin 28B rs12979860 C/T polymorphism on the histological outcome of chronic hepatitis C: relationship with gender and viral genotype. J Clin Immunol.

[16] Ghany MG, Strader DB, Thomas DL, Seeff LB (2009). Diagnosis, management, and treatment of hepatitis C: an update. Hepatology.

[17] El Awady MK, Bader El Din NG, Tabll A, El Hosary Y, Abdel Aziz AO, El Khayat H, Salama M (2013). IL28B polymorphism and cytomegalovirus predict response to treatment in Egyptian HCV type 4 patients. World J Gastroenterol.

[18] Tozun N, Ozdogan OC, Cakaloglu Y, Idilman R, Karasu Z, Akarca U (2010). Nationwide prevalence study and risk factors for hepatitis A, B, C and D infections in Turkey. Hepatology.

[19] Ozer B, Seydaoglu G, Ozsahin AK, Demirhindi H (2012). Risk factors for higher anti-HCV positivity in a border city in southern Turkey with unique population characteristics. Turk J Gastroenterol.

[20] Yildirim B, Barut S, Bulut Y, Yenisehirli G, Ozdemir M, Cetin I, Etikan I (2009). Seroprevalence of hepatitis B and C viruses in the province of Tokat in the Black Sea region of Turkey: A population-based study. Turk J Gastroenterol.

[21] Gokahmetoglu S, Atalay MA, Kilinc A (2011). Determination of the hepatitis C virus genotypes with "pyrosequencing" method. Erciyes Medical Journal.

[22] Manolio TA (2010). Genomewide association studies and assessment of the risk of disease. N Engl J Med.

[23] Kelly C, Klenerman P, Barnes E (2011). Interferon lambdas: the next cytokine storm. Gut.

[24] El-Awady MK, Mostafa L, Tabll AA, Abdelhafez TH, Bader El Din NG, Zayed N, Shenawy RE (2012). Association of IL28B SNP With Progression of Egyptian HCV Genotype 4 Patients to End Stage Liver Disease. Hepat Mon.

[25] Liao XW, Ling Y, Li XH, Han Y, Zhang SY, Gu LL, Yu DM (2011). Association of genetic variation in IL28B with hepatitis C treatment-induced viral clearance in the Chinese Han population. Antivir Ther.

[26] Ruiz-Extremera A, Munoz-Gamez JA, Salmeron-Ruiz MA, de Rueda PM, Quiles-Perez R, Gila-Medina A, Casado J (2011). Genetic variation in interleukin 28B with respect to vertical transmission of hepatitis C virus and spontaneous clearance in HCV-infected children. Hepatology.

[27] Fabris C, Falleti E, Cussigh A, Bitetto D, Fontanini E, Bignulin S, Cmet S (2011). IL-28B rs12979860 C/T allele distribution in patients with liver cirrhosis: role in the course of chronic viral hepatitis and the development of HCC. J Hepatol.

[28] Pasha HF, Radwan MI, Hagrass HA, Tantawy EA, Emara MH (2013). Cytokines genes polymorphisms in chronic hepatitis C: impact on susceptibility to infection and response to therapy. Cytokine.

[29] McHutchison JG (2011). The role of genetic markers in hepatitis C virus therapy: a major step for individualized care. Liver Int.

[30] McCarthy JJ, Li JH, Thompson A, Suchindran S, Lao XQ, Patel K, Tillmann HL (2010). Replicated association between an IL28B gene variant and a sustained response to pegylated interferon and ribavirin. Gastroenterology.

[31] Montes-Cano MA, Garcia-Lozano JR, Abad-Molina C, Romero-Gomez M, Barroso N, Aguilar-Reina J, Nunez-Roldan A (2010). Interleukin-28B genetic variants and hepatitis virus infection by different viral genotypes. Hepatology.

[32] Tanaka Y, Nishida N, Sugiyama M, Kurosaki M, Matsuura K, Sakamoto N, Nakagawa M (2009). Genome-wide association of IL28B with response to pegylated interferon-alpha and ribavirin therapy for chronic hepatitis C. Nat Genet.

[33] Liu CH, Liu CJ, Lin CL, Liang CC, Hsu SJ, Yang SS, Hsu CS (2008). Pegylated interferon-alpha-2a plus ribavirin for treatment-naive Asian patients with hepatitis C virus genotype 1 infection: a multicenter, randomized controlled trial. Clin Infect Dis.

[34] Thompson AJ, Muir AJ, Sulkowski MS, Ge D, Fellay J, Shianna KV, Urban T (2010). Interleukin-28B polymorphism improves viral kinetics and is the strongest pretreatment predictor of sustained virologic response in genotype 1 hepatitis C virus. Gastroenterology.

[35] Sarrazin C, Schwendy S, Moeller B, Dikopoulos N, Buggisch P, Encke J (2010). Completely individualized treatment durations with peginterferon-alfa-2b and ribavirin in HCV genotype 1-infected patients and importance of IL28B genotype (INDIV-2 study). Hepatology.

[36] Stattermayer AF, Stauber R, Hofer H, Rutter K, Beinhardt S, Scherzer TM, Zinober K (2011). Impact of IL28B genotype on the early and sustained virologic response in treatment-naive patients with chronic hepatitis C. Clin Gastroenterol Hepatol.

[37] Shaker OG, Sadik NA (2012). Polymorphisms in interleukin-10 and interleukin-28B genes in Egyptian patients with chronic hepatitis C virus genotype 4 and their effect on the response to pegylated interferon/ribavirin-therapy. J Gastroenterol Hepatol.

